# Cross‐Linked Hyaluronic Acid and Laser Photobiomodulation in Guided Bone Regeneration (GBR): A Case Report Describing a New Combined Technique

**DOI:** 10.1155/crid/7424509

**Published:** 2026-02-11

**Authors:** Paolo Vescovi, Ilaria Giovannacci, Carmen Mortellaro, Alberta Greco Lucchina, Roberta Iaria, Gloria Bortolotti, Jair Carneiro Leão, Marco Meleti, Samir Nammour

**Affiliations:** ^1^ Department of Medicine and of Surgery, University of Parma, Parma, Italy, unipr.it; ^2^ UniCamillus, Saint Camillus International University of Health Sciences, Rome, Italy, unicamillus.org; ^3^ Department of Clinical and Preventive Dentistry, Federal University of Pernambuco (UFPE), Recife, Pernambuco, Brazil, ufpe.br; ^4^ Department of Dental Sciences, Faculty of Medicine, University of Liege, Liege, Belgium, ulg.ac.be

**Keywords:** cross-linked hyaluronic acid, esthetic area rehabilitation, guided bone regeneration, Nd: YAG laser, porcine pericardium membrane

## Abstract

Perfect maintenance of the soft tissue and bone level at the implant site substantially influences the long‐term surgical success and esthetic and functional results of prosthetic rehabilitation. Hyaluronic acid (HA) is a glycosaminoglycan, that is a long linear polymer chain formed by the repetition of disaccharide units, in turn composed of alternating molecules of glucuronic acid and N‐acetylglucosamine joined together by glycosidic and hydrogen bonds. HA in oral surgery improves wound healing by stimulating clot formation, inducing angiogenesis, increasing osteogenesis, preserving the viability of periodontal ligament fibroblasts and gingival fibroblasts, and accelerating bone regeneration through chemotaxis, proliferation, and subsequent differentiation of mesenchymal cells. The cross‐linking process, which modifies the three‐dimensional structure of the HA chains, gives the product a higher molecular weight and, consequently, a greater density, slower reabsorption, and longer lasting action. Laser photobiomodulation (laser PBMT) promotes wound healing by inducing cell proliferation, anti‐inflammatory responses, pain relief, and scar formation inhibition. The present paper describes the clinical case of a 72‐year‐old male patient who, due to a large vestibular bone defect in Zone 1.1, required bone regeneration. The aim of this case report was to propose a combination of cross‐linked hyaluronic acid (xHyA) and laser PBMT to support bone and soft tissue regeneration in areas of severe periodontal compromise and tissue defects.

## 1. Background

Esthetic results are currently recognized as one of the determining factors that influence the success of implant rehabilitation and patient satisfaction. It is well established that functionality and esthetics are closely linked; the perfect maintenance of the level of soft tissue and bone at the implant site substantially influences the esthetic and functional results of the implant [1].

Postextractive implant placement with immediate loading rehabilitation can be an esthetically successful intervention. It represents a less traumatic procedure, significantly reduces the number of surgical interventions, and ensures an improvement in the patient′s quality of life. The lack of bone or covering tissue sometimes makes it impossible to position the implant, and guided tissue regeneration (GTR) interventions are necessary.

The presence of bone defects at the implant site, chosen according to the prosthetic project, poses a challenge for clinicians, especially when they affect an aesthetic area. Several studies in the currently available literature evaluate the effectiveness of different biomaterials in restoring bone quantity and quality.

Many surgical and medical techniques, as well as the use of different technologies, have been proposed in the literature to optimize implant success in aesthetic areas [2–5].

Hyaluronic acid (HA) is a macromolecular anionic polymer that exists in the human body; is widely found in connective, epithelial, and nervous tissues; and is widely used in medicine, cosmetics, and other biomedical applications.

HA is identified as a glycosaminoglycan, which is a long linear polymer chain formed by the repetition of disaccharide units, composed in turn by the molecular alternation of glucuronic acid and N‐acetylglucosamine, joined together by glycosidic and hydrogen bonds. This structure makes HA capable to retain large amounts of water and provide hydration to the connective tissues of the human body. Its presence guarantees, at the skin level, elasticity, softness, brightness and hydration to the face and body skin, and protects it from damage caused by UV rays, external factors such as smog, and physical stress. HA present in the tissues is continuously metabolized and eliminated.

HA has moisturizing, repairing, and regenerative properties, depending on its molecular weight. The hygroscopic proprieties of HA are conferred by its neutral pH. This allows it to attract large quantities of water molecules; indeed, it is capable of retaining up to 10,000 times its weight in water. These characteristics explain the role of HA in maintaining the water balance of tissues. This prerogative, together with its unique viscoelastic and physicochemical properties, has led to the development of numerous medical devices based on HA with excellent biocompatibility, low toxicity, and full biodegradability in vivo [6].

The half‐life of HA does not exceed 48 h in the balance between its production and demolition by hyaluronidase. To overcome the short half‐life of endogenous HA in regenerative medicine and surgery, chemical modifications are required to obtain products that are more resistant to demolition and therefore a longer duration. The linear form of HA was the first to be commercialized and was made of unmodified HA chains. Cross‐linked hyaluronic acid (xHyA) is a synthetic form of the molecule, characterized by cross‐links between several molecules of linear HA, obtained during the productive phases [7]. These cross‐links are characterized by transversal bridges connecting multiple linear HA molecule. As a result, xHyA is larger in size and has a higher molecular weight and therefore greater density, a characteristic that promotes slower resorption and thus longer lasting action [8].

Wound healing (traumatic or surgical) involves a series of consequential and partly overlapping phases, including a hemostasis/inflammation phase, proliferation phase, and remodeling phase. Fibrinogen is deposited by platelets during the initial clot formation. Fibrinogen is an HA‐binding protein and, together with the resulting fibrin, contributes to clot formation and the maintenance of the local HA concentration. HA acts as a structural matrix for coagulated fibrin deposition.

HA in oral surgery shows physicochemical and biological properties, such as hygroscopic, viscoelastic, bacteriostatic, anti‐inflammatory, and antiedematous properties, and improves bone formation.

In particular, HA in guided bone regeneration (GBR) significantly stimulates clot formation, induces angiogenesis, increases osteogenesis, preserves the vitality of periodontal ligament and gingival fibroblasts, and increases the proliferative capacity and migration of both cell types. HA accelerates bone regeneration by chemotaxis, proliferation, and subsequent differentiation of mesenchymal cells. HA shares bone‐inducing characteristics with osteogenic substances such as bone morphogenetic protein 2 and osteopontin [9, 10].

Photobiomodulation therapy (PBMT) is defined as light therapy using low‐level lasers and LEDs that promotes wound healing by inducing cell proliferation, anti‐inflammatory response, pain relief, and inhibition of scar formation. Many in vitro and in vivo studies have been performed to clarify the utility of lasers as a tool to stimulate cell proliferation and wound healing. Some authors agree that low‐power laser irradiation (those that provide an output on the order of milliwatts) has an important role in triggering cell proliferation and in treating diseases of various origins [11]. PBMT represents a promising approach for the management of mucosal defects caused by inflammatory conditions and autoimmune lesions [12]. Many studies have demonstrated the osteoinductive capabilities of different laser wavelengths, inducing both osteoblast proliferation and differentiation, with increased cellular activity. In vivo experimental models demonstrate that laser irradiation after surgical bone defects (tooth extractions, osteotomies, and dental implants) can promote osteoblast differentiation and increased expression of osteocalcin (OCN), matrix metalloproteinases (MMPs), and other markers of bone metabolism [13–15].

Several in vitro studies have been carried out to clarify the effectiveness of laser PBMT as a bactericidal effect and as a stimulus for cell proliferation and wound healing. Lymphatic and blood vessels also appeared to be stimulated by laser irradiation. Molecular factors such as light absorption by mitochondrial enzymes, cytochromes, flavins, and porphyrins may also stimulate [16].

Lasers have been widely used in periodontal and endodontic treatments because of their proven bactericidal activity. Treatment of periodontal pockets and advanced peri‐implantitis also appears to improve by reinforcing conventional treatment with laser PBMT [17–19].

The aim of the present case report was to propose a combination of xHyA mixed to particulate deantigenated bovine bone and laser PBMT to support bone and soft tissue regeneration in areas of severe periodontal impairment and tissue defects. In particular, the main purpose is to combine the regenerative effects of xHyA and the biostimulating effects of laser photobiomodulation, in order to optimize the regenerative potential and the clinical outcomes.

## 2. Methods

The clinical case reported in this paper was conducted in accordance with the principles of the Declaration of Helsinki. The patient signed an informed consent form for treatment, agreeing to undergo surgery as described below.

**Figure 1:**
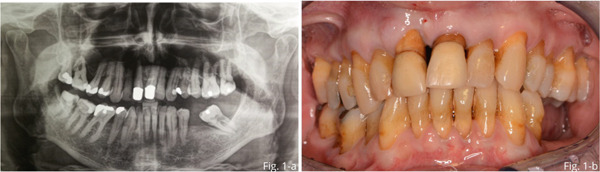
(a) Front in occlusion. (b) Patient OPT of the baseline situation.

The case involved a 72‐year‐old male patient who presented to our clinic with a periodontal abscess of the central incisor. The element was periodontally compromised and presented a vertical fracture of the root (PPB = 10 mm; BOP: +) and could not be recovered (Figure 1a,b). Since the patient presented severe infection, antibiotics were prescribed (amoxicillin 1 g and metronidazole 500 mg every 12 h) for 5 days. At the end of the medical therapy for infection resolution, the extraction of the tooth and the GBR treatment to fill the buccal bone defect in view of a subsequent implant insertion were performed.

**Figure 2:**
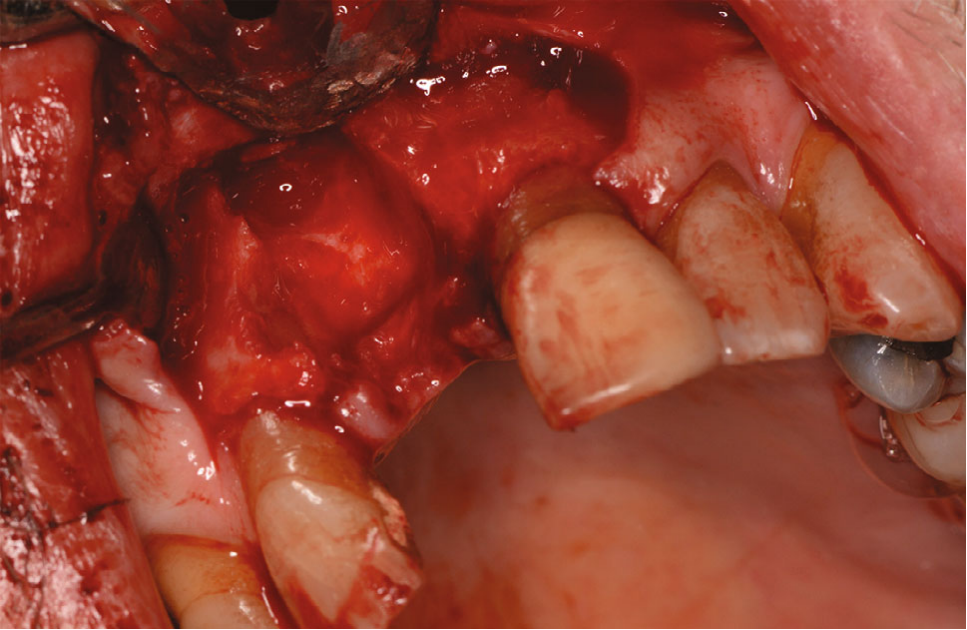
Postextraction socket and exposure of vestibular bone defect.

Following locoregional anesthesia, a trapezoidal flap was created to expose the vestibular bone defect (Figure 2), extraction of the element and curettage of the alveolus.

**Figure 3:**
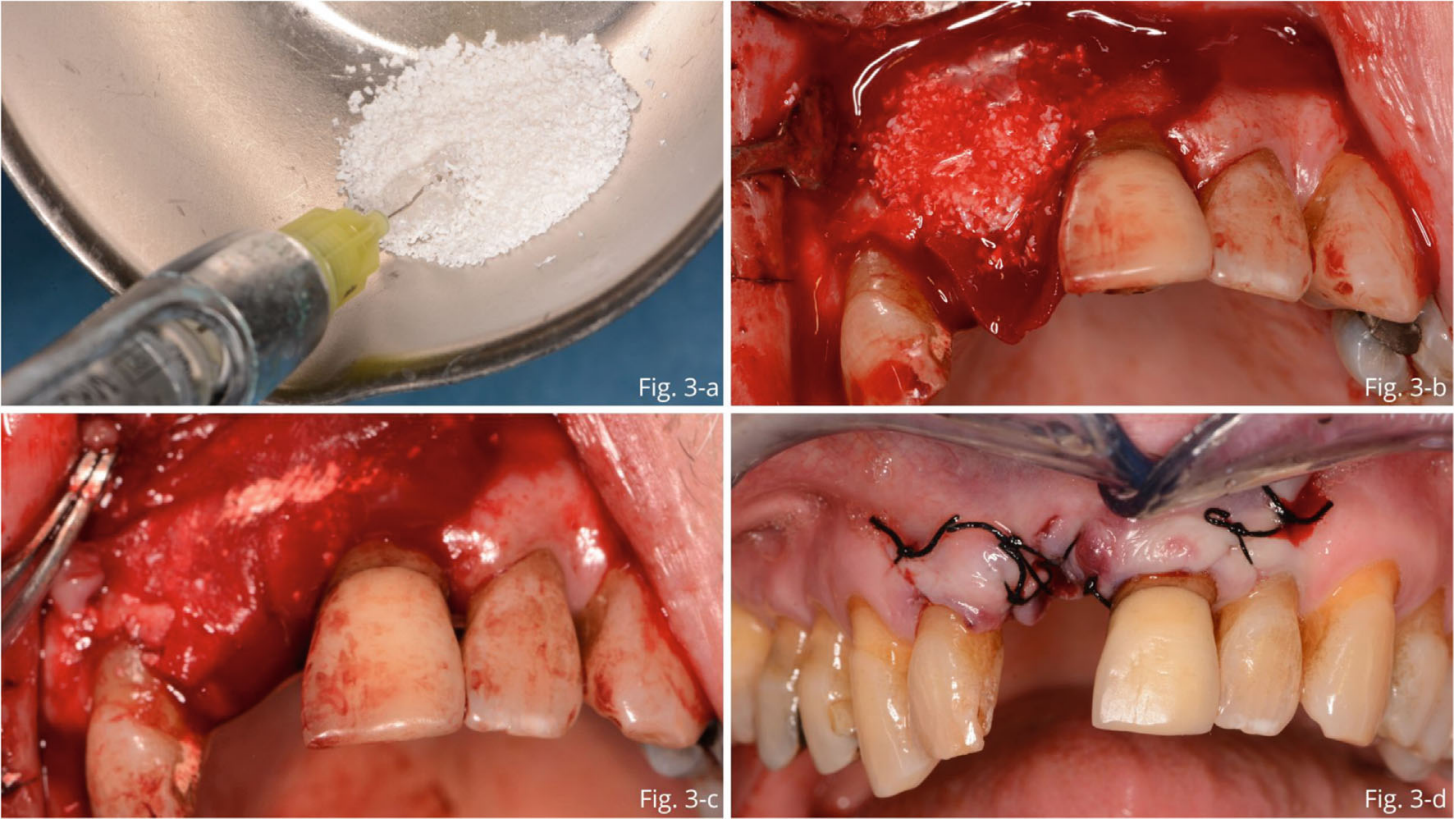
(a) Particulate deantigenated bovine bone mixed with cross‐linked hyaluronic acid gel (HA). (b) Bone defect filled with mixture of xHyA gel and particulate deantigenated bovine bone. (c) Application of a collagen membrane to protect the graft material. (d) Silk 4/0 single sutures.

Subsequently, particulate deantigenated bovine bone (Bio‐Oss, Geistlich, Switzerland) was mixed with xHyA gel (hyaDENT BG, Regedent AG, Zurich, Switzerland): composition: 1.6% xHyA, 0.2% native HA (Figure 3a) and placed to fill the bone gap (b). A resorbable collagen membrane was applied to protect the graft material (Figure 3c). Lastly, to achieve healing by first intention, the flap was released and sutured using a single nonabsorbable 4/0 silk (Figure 3d).

**Figure 4:**
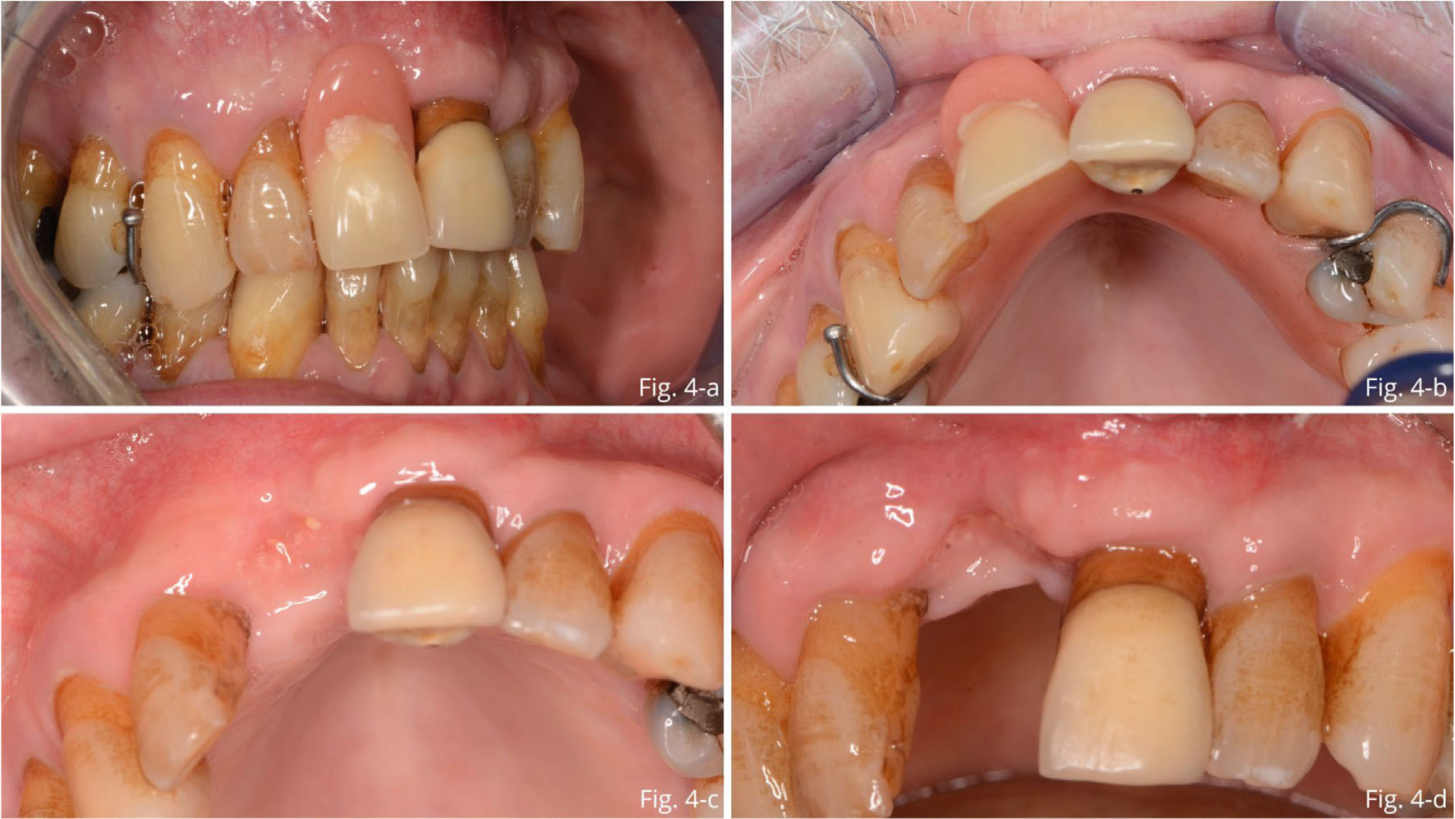
(a, b) Removable temporary prosthesis fitting. (c, d) 4 months follow‐up.

After a week, the sutured were removed, and a temporary prosthesis was provided (Figure 4a,b). At 4 months, there was complete mucosal healing without signs of infection or inflammation (Figure 4c,d). Seven months after surgery, the success of regenerative surgery with bone formation at site 1.1 was observed by CT scan, and implant placement was planned. The CT scan also revealed a periapical lesion on 2.1, an element that had already been treated endodontically and covered with a ceramic crown (Figures 5a, 5b, 5c, and 5d).

Figure 5The CT scan shows the success of regenerative surgery and the presence of a periapical lesion at the 2.1 level. (a) Coronal CT slice. (b) Three‐dimensional reconstruction of the coronal slice. (c) Occlusal view of the three‐dimensional scan reconstruction. (d) Measurement of bone thickness calculated on sagittal slices.

**Figure:**
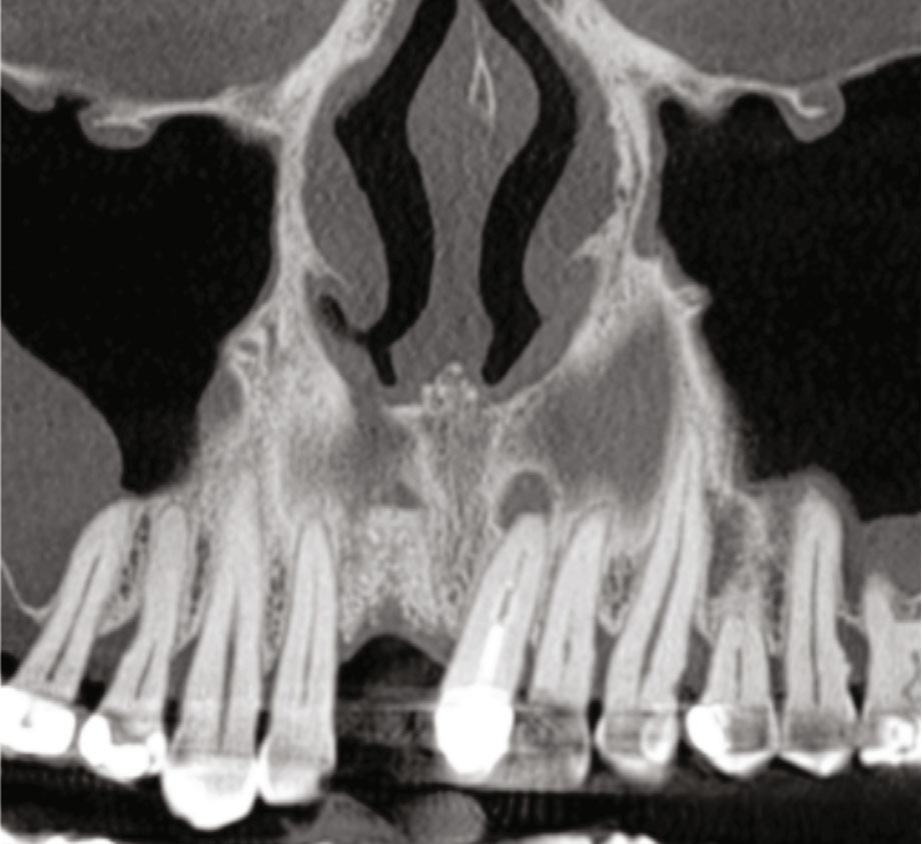
(a)

**Figure:**
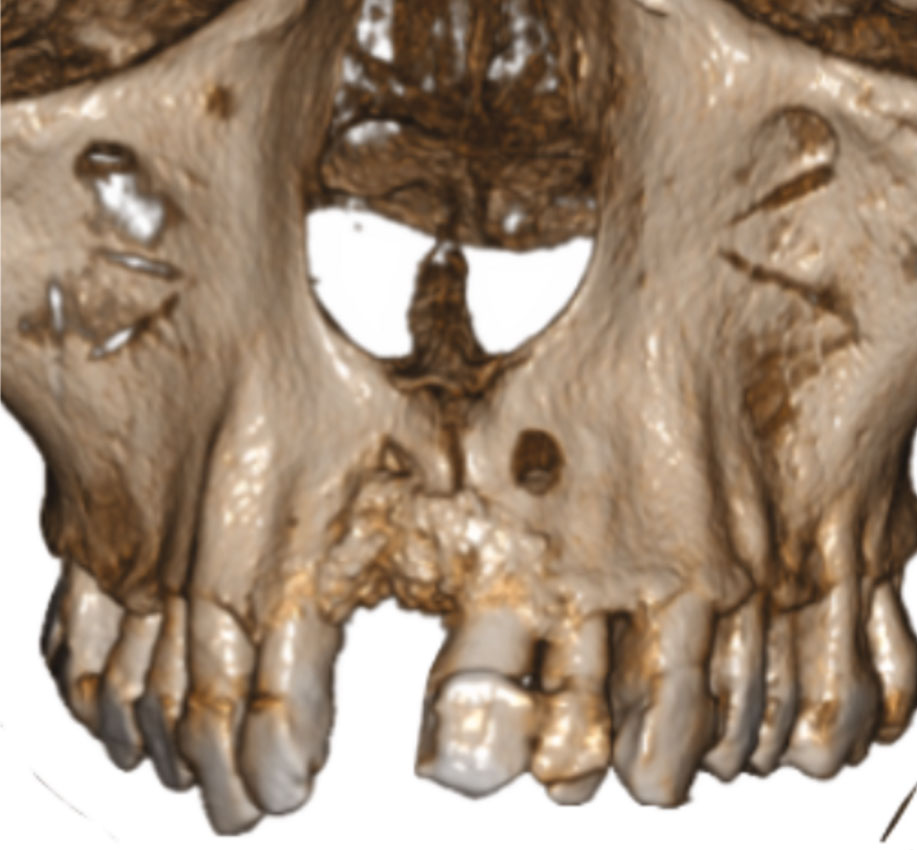
(b)

**Figure:**
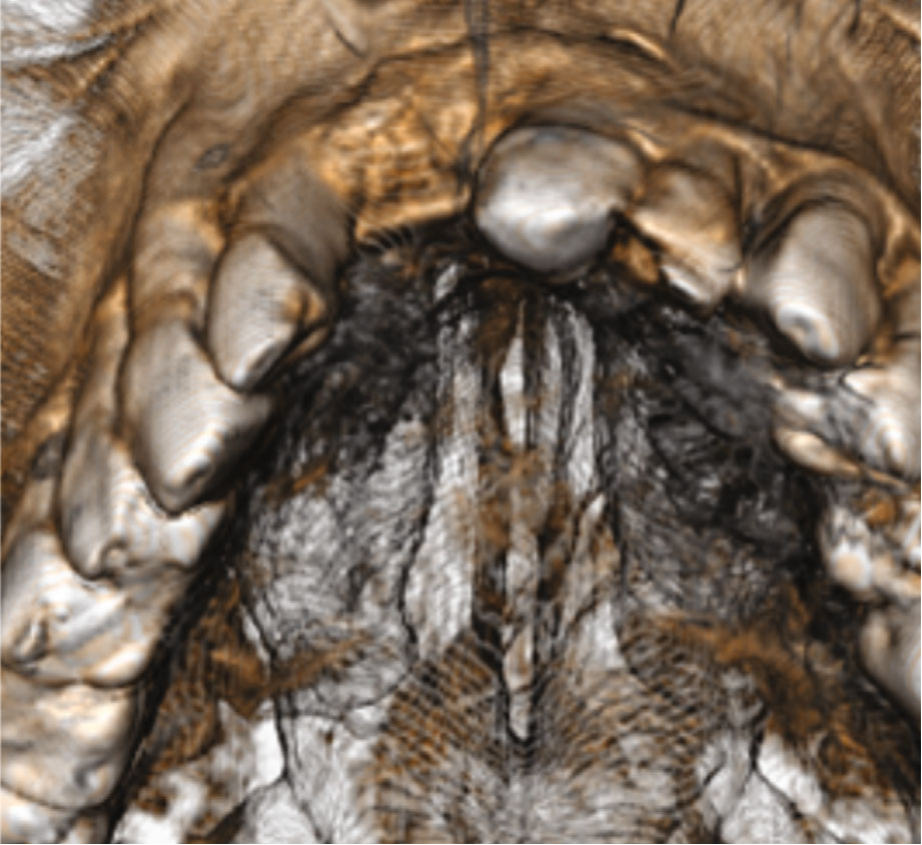
(c)

**Figure:**
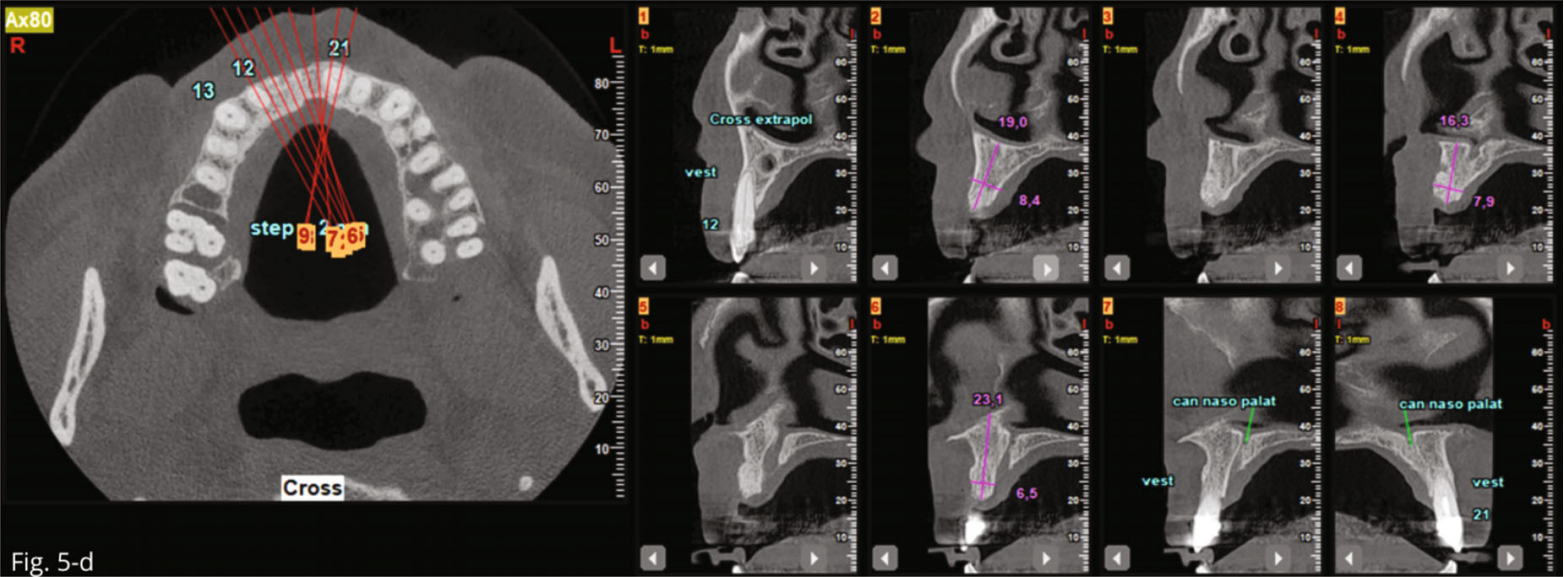
(d)

**Figure 6:**
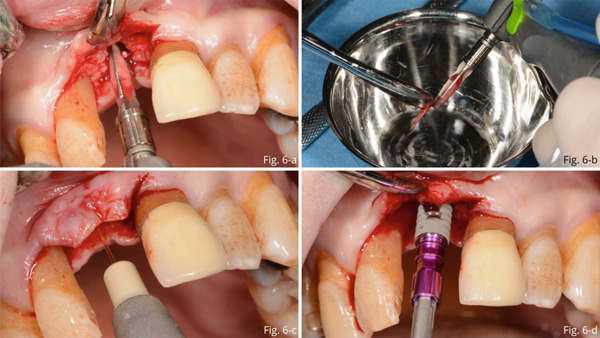
(a) Preparation of the implant cavity. (b) Retrieving of autologous bone chips retained between drills blades. (c) Photobiomodulation with Nd:YAG laser. (d) Implant placement.

Ten months later, implant placement was performed at Site 1.1. Under local anesthesia, a crestal incision was made and the alveolar ridge was exposed. The cavity was then prepared for implant placement and autologous bone chips retained between the blades of the drills were retrieved (Figure 6a,b).

Subsequently, the site was irrigated with iodopovidone and photobiostimulated (Figure 6c) using a Neodymium laser (Nd:YAG laser 1064 nm; 320 microns fiber, 1 W power, and 10 Hz frequency for 5 min) inserting the fiber in such a way as to allow irradiation of the bone surface.

After these steps, implant placement (4.3 × 13 mm) was performed (Figure 6d).

**Figure 7:**
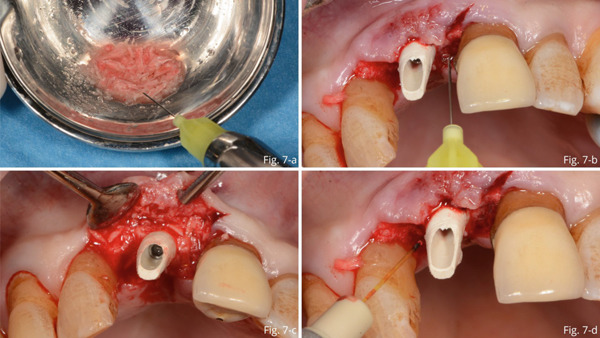
(a) Autologous graft material mixed with xHyA gel. (b) The residual gap between the abutment and the gingiva filled with the direct application of hyaluronic acid. (c) Placement of additional autologous bone and xHyA mixture. (d) Photobiomodulation with Nd:YAG laser.

**Figure 8:**
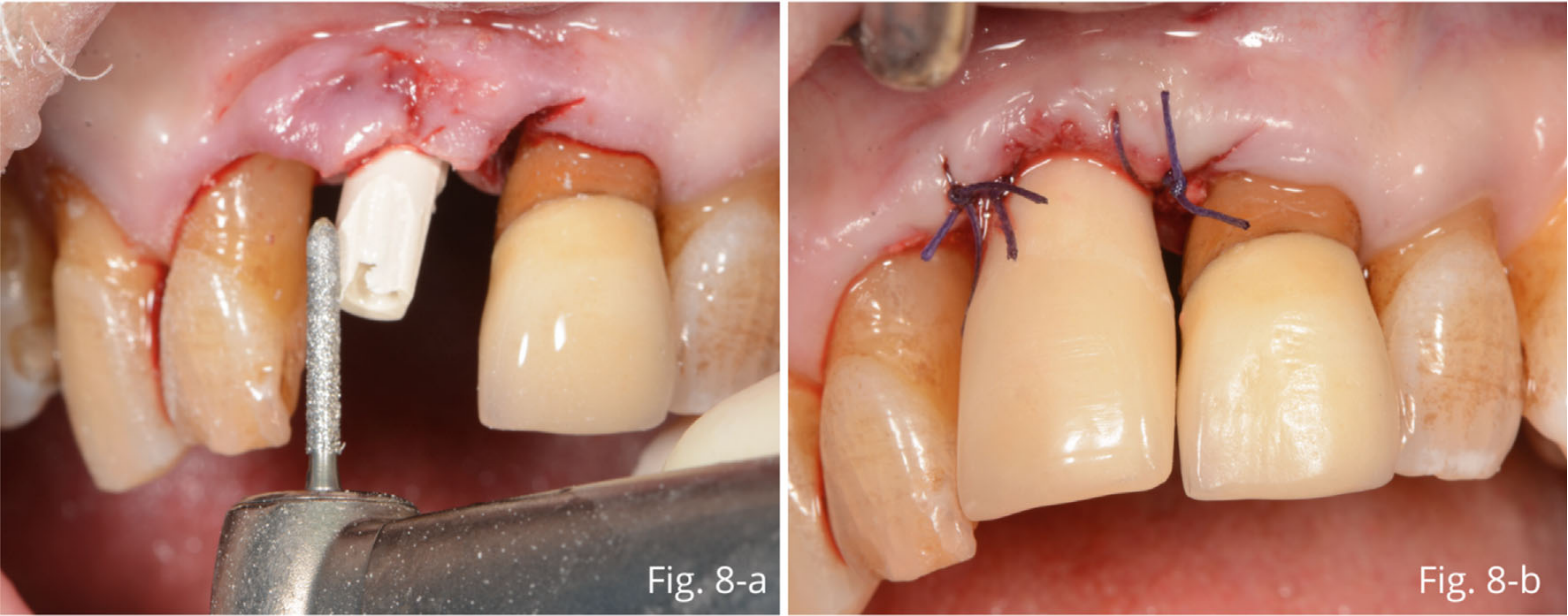
(a) Insertion and preparation of the temporary abutment. (b) Silk 4/0 single sutures.

Autologous graft material was mixed xHyA gel and placed at the level of the bone gap (Figure 7a). After insertion and preparation of the temporary abutment, the residual gap between the abutment and the gingiva was filled with the direct application of further gel (Figure 7b) and the placement of additional autologous bone and xHyA mixture (Figure 7c). Then, laser PBMT was performed using Nd:YAG (Figure 7d). The temporary crown was relined and cemented onto the abutment (Figure 8a,b).

**Figure 9:**
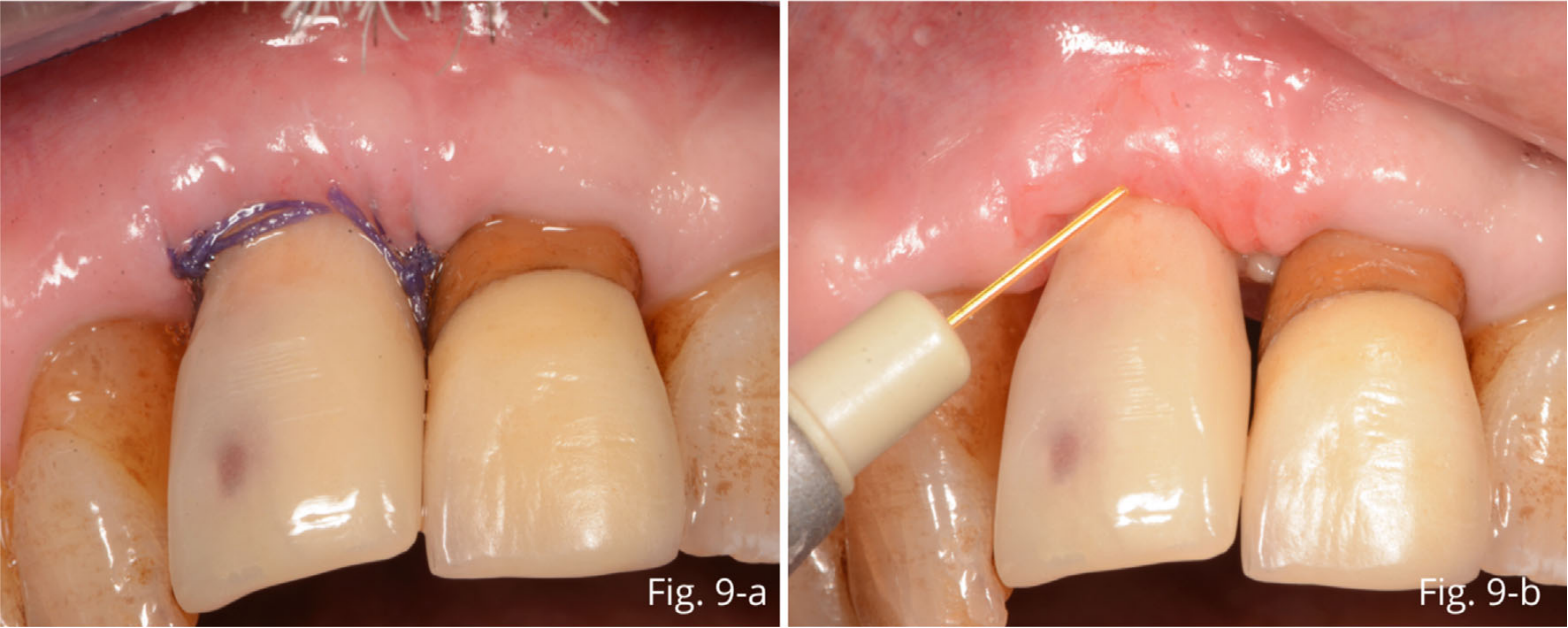
(a, b) 2‐week follow‐up.

At the first follow‐up visit 2 weeks after surgery, the healing of the gingival tissue in the complete absence of inflammation was observed (Figure 9a). The sutures were removed and laser photobiostimulation with Nd:YAG was performed (Figure 9b). Subsequently, the crown of Tooth 2.1 was removed, and root canal retreatment was performed.

## 3. Results

**Figure 10:**
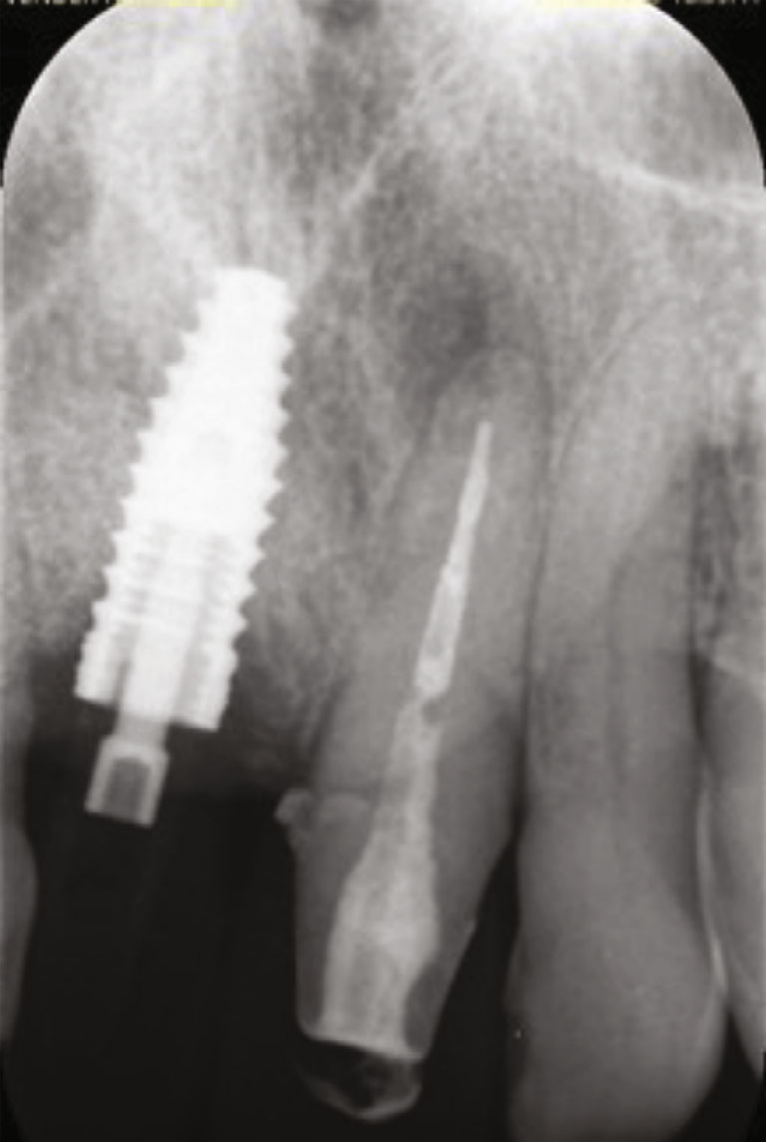
Four months X‐ray control.

At 4 months, an endoral periapical control X‐ray was taken, in which implant osseointegration was observed. In addition, almost complete healing of the periapical lesion of 2.1 could be seen on the X‐ray (Figure 10).

**Figure 11:**
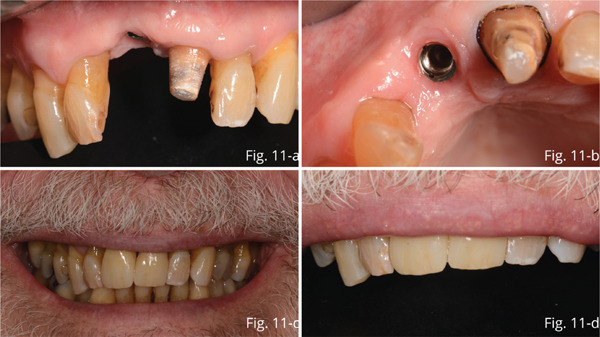
(a) Gingival profile with good maintenance of thickness, in the absence of inflammation. (b) Modeling of the gingival tunnel around the implant in the absence of inflammation. (c) Full smile prosthetic result. (d) Final result of the incisal edge.

Five months after surgery, uniform healing and good modeling of the gingival tunnel and parabola were evident (Figure 11a,b).

In addition, the 2.1 abutment was prepared, and scans of the latter and the 1.1 implant were taken. Then, the color was chosen, and after 20 days, the case was finalized: A zirconia abutment was fabricated on the implant on which a zirconia crown was cemented, while a zirconia crown was cemented on the 2.1‐tooth abutment using a dual‐curing resin cement (Panavia V5).

The prosthetic restorations were made in such a way as to integrate perfectly with the patient′s periodontal and dental situation to achieve as natural an effect as possible. From the photos, it is possible to observe a healing both of hard and soft of the tissues, which led to a clear improvement of the gingival profile compared to the starting situation (Figure 11c,d).

**Figure 12:**
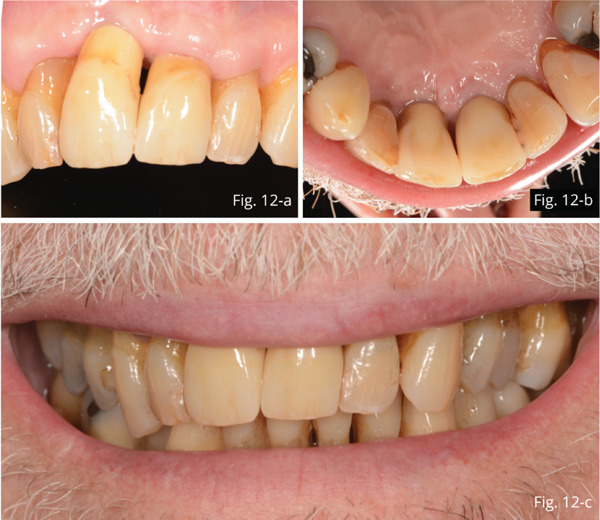
(a–c) 12‐month follow‐up.

Twelve months later, efficient maintenance from a functional point of view and tissues in the absence of inflammation and discomfort are observed. The patient was satisfied with both aesthetics and function (Figures 12a, 12b, 12c, and 12d).

**Figure 13:**
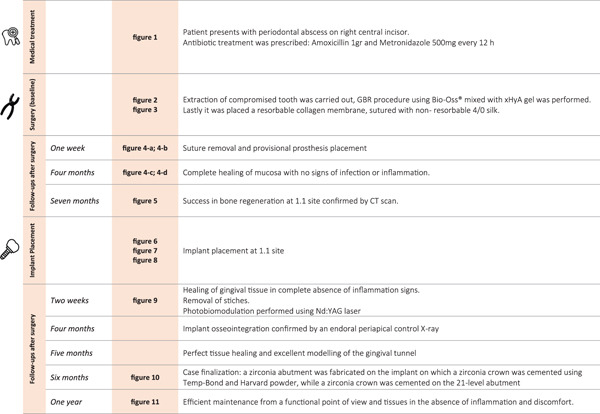
Flowchart summarizing treatment and follow‐up history of the patient.

Figure 13 reports all the stages of treatment and follow‐ups carried out on the patient.

## 4. Discussion

Periodontal or bone regeneration can be defined according to the type of tissue involved: GTR and GBR. The term GTR refers to the regeneration of the periodontal attachment, including the bone and cementum, while GBR generally refers to bony defects in edentulous areas that are likely to be subject to implant rehabilitation. In GBR, heterologous materials, autologous grafts of bone and mucous tissue, and technologies that promote regenerative biological processes such as lasers are used.

Alveolar ridge augmentation using GBR results in predictable bone formation with high long‐term success rates. Bioresorbable membranes are frequently used for in GBR [20]. The membranes act as barriers against the ingrowth of epithelial and connective tissue cells into the defect. Furthermore, they stabilize the blood clot and prevent flap collapse, thus maintaining the space needed for bone tissue regeneration. Membrane resorption occurs through a biodegradation process that begins as cells within the surgical site release MMPs into the wound area during healing and continues with the infiltration and colonization of fibroblasts and blood vessels, leading to membrane degradation.

MMPs represent a major group of enzymes that regulate cell–matrix composition. MMPs are produced by a number of cell types, including fibroblasts, macrophages, endothelial cells, and keratinocytes [21–23].

After tooth extraction, the alveolar ridge undergoes substantial horizontal and vertical resorptions. These dimensional changes are clinically relevant as they can impede the placement of dental implants and compromise soft tissue aesthetics. Alveolar ridge preservation (RAP) is based on the placement of a bone‐replacement graft in the extraction socket to reduce horizontal and vertical resorption of the alveolar process after extraction.

The available literature provides numerous studies investigating the combination of HA with various scaffolds and materials used in implantology (from calcium phosphate salts to certain minerals, as well as inorganic and organic polymers) to promote bone deposition [23]. These materials are limited by uncertain degradation rates; therefore, strategies combining ceramics and polymers such as HA have been developed to address these deficiencies. Traditional scaffolds typically lack osteoinductive properties. Therefore, the introduction of osteoinductive or osteogenic components via a delivery system can be considered an enhancement of intrinsic scaffolds [24–27].

Preclinical studies have investigated the rate of bone regeneration in defect resulting from surgery. The findings show that when autogenous bone grafts, collagen sponges or xenografts are combined with HA, there is an increase in osteogenesis and a lower amount of residual material at the surgical site.

De Brito Bezerra et al. conducted a study concluding that the combination of a 1% HA and an absorbable collagen sponge (ACS) result in improved new bone formation in critical‐sized calvaria defects in rats [28].

Arpag et al. conducted a study to determine bone‐healing capacity of high molecular weight HA combined with xenograft in rabbit calvaria bone defects. Their results suggest that high molecular weight HA contributes positively to the healing of xenografts by increasing the percentage of new bone tissue formation and reducing the residual graft quantity. However, in the same study, the analysis of microarchitectural parameters showed that HA did not significantly affect the quality of newly formed bone tissue, influencing only the quantitative aspect [29].

Lee et al. conducted a study to evaluate the efficacy of deproteinized bovine bone mineral with 10% collagen (DBBM‐C) soaked in HA for ridge preservation in compromised extraction sockets. The results showed that ridge preservation with the DBBM‐C/HA mixture prevented shrinkage and improved bone formation in the compromised extraction sockets at 1 and 3 months [30].

In the present case, after tooth extraction, to cover the large bone defect, we used a resorbable collagen membrane soaked in HA and inserted particulate deantigenated bovine bone mixed with HA. During and after the surgical procedure, we performed laser PBM with a Nd:YAG laser (1064 nm). After 4 months, despite the total loss of the buccal cortex and part of the lingual wall and severe loss of gingival height and profile, we obtained a perfect maintenance of alveolar volume for both soft tissues and bone.

Several studies have been conducted to evaluate the osteogenic potential of HA in implantology. Notably, the osteoinductive properties of HA, combined with the stimulation of bone tissue progenitor cells, appear to be the determining factors behind secondary stability [31].

A recent study conducted by Scarano et al. in 2025 evaluated the effectiveness of HA enriched with amino acids in osteogenesis. Results from the same study demonstrate that the application of these biomaterials into bone defects enhances bone formation, angiogenesis, and osteoblastic count [32].

Zou et al. observed that 800‐kDa HA added to bone marrow stromal cells cultured in vitro accelerates cell proliferation, increases cellular alkaline phosphatase (ALP) activity and OCN gene expression, and that HA interacts with human bone morphogenic protein‐2 (BMP‐2) to generate direct and specific cellular effects [31].

Itoh and co‐workers conducted a study with the aim of evaluating the efficacy of high molecular weight HA used as a carrier of recombinant human bone morphogenetic protein‐2 (rhBMP‐2) adsorbed to a titanium fiber mesh implant (TFMI) in vivo. The results showed that high‐molecular‐weight HA is not only a carrier of BMP but also has a positive effect on the generation of new bone in TFMIs. [33]

HA has the potential to be a valuable healing agent for this indication owing to its bacteriostatic, anti‐inflammatory, and immunosuppressive properties. As HA is also involved in various signaling pathways that are activated during wound healing, it may facilitate re‐epithelization.

Hasan et al. evaluated the effects of HA on the bone‐implant interface in rabbits by immunohistochemical estimation of tumor necrosis factor alpha (TNF‐*α*) in experimental and control groups. The results revealed a positive localization of TNF‐*α* by bone marrow stromal cells, especially in the 2‐week healing period, and by bone cells, including osteoblasts, osteocytes, and osteoclasts, at different intervals in both groups, with higher scores in the HA‐coated group at almost all healing intervals compared to the control group [34]. The conclusions of the aforementioned study support that HA can effectively promote osteoconduction and therefore the osseointegration process, an imperative for successful implant placement [34].

Several clinical studies have confirmed the clinical, radiological, and histological analyses of the effects of xHyA on periodontal wound healing. HA has demonstrated successful results in terms of clinical attachment level gain and probing depth reduction in animal models and in humans [35–40].

Recently, it was reported that xHyA when applied to the gingival sulcus during nonsurgical periodontal therapy induces positive effects on cells involved in periodontal wound healing, thus pointing to its potential use in nonsurgical periodontal therapy [31, 35]. The literature supports the use of xHyA in soft tissue regeneration, particularly in the maintenance of gingival volume and profile.

HA is a physiologically present component in all stages of bone and soft tissue healing and plays many different roles, especially in the early stages of defect repair. In GBR, xHyA showed positive effects. Through the induction of osteogenic cell differentiation, bone can accelerate the osteogenic process during wound healing and promote osseointegration of implants, as well as soft tissue healing.

It is widely reported in the literature that laser PBMT has biostimulatory, anti‐inflammatory, and analgesic effects that can be useful in periodontal and implant treatment procedures [41]. A recent review of the literature confirms the positive effect of laser PBMT on the stimulation of healing of periodontal soft and hard tissues with a significant reduction of inflammation and postoperative pain [42, 43].

In the second intervention, at the time of implant placement, we combined HA with the patient′s native bone, collected by the drill during the preparation of the implant site, to fill the small residual defects. Laser application was performed immediately after site preparation before to insert the implant and at the end of surgical procedure HA was inserted in contact with the implant surfaces and under the gingival flap in contact with the particulate bone.

A recent meta‐analysis confirmed that additional PBMT was performed with laser wavelengths ranging from 618 to 1064 nm. It can be safely prescribed after the surgical placement of titanium implants without adverse events and with clinical or biological benefits. Most experiments have reported statistically significant improvements in the implant stability. The time taken to deliver laser PBMT was not a variable that differentiated whether a study reported significant results [44]. Randomized clinical trials have reported the positive effect of laser PBMT on bone and mucous tissue to reduce inflammation and improve early healing [45, 46].

Several experiments in animal models have reported a positive effect of the laser PBMT on implant bone healing. Histological and histomorphometric analyses indicated that the irradiated groups showed faster production of bone tissue matrix with newly formed bone areas than the control groups [47, 48].

Stabilization of dental implants (primary and secondary) and bone density in the peri‐implant area after laser application have been widely reported in the literature. In general, the implant stability quotient (ISQ) was assessed at both irradiated and nonirradiated sites immediately after surgery, as well as in the postoperative period ranging from 1 to 3 months. Significant differences were reported between the ISQ values at the irradiated sites immediately after surgery, up to the prosthetic masticatory load. Laser PBMT resulted in a favorable increase in the ISQ value at 3 months [49, 50, 51].

Laser PBMT has been extensively studied and its biomodulatory effects have been established on irradiated cells, increasing viability and proliferation, and on damaged tissues. Furthermore, laser PBMT can reduce and modulate the inflammatory process and improve the postoperative healing period in terms of pain, trismus, and edema.

## 5. Conclusion

Laser PBMT combined with the use of xHyA and biomaterials could be considered as a way to enhance the regeneration of bone and mucosal defects and promote osseointegration and implant success. Moreover, the bone graft material used in the case described comes in the form of microgranules, with its own three‐dimensional structure that creates a scaffold for the growth of new bone tissue, maintaining volume and allowing it to be replaced by native bone tissue over time. xHyA supports bone regeneration as it also creates a three‐dimensional scaffold that stabilizes the clot, attracting the necessary growth factors.

Despite the promising results of the surgical technique described in this paper, studies on a larger statistical population would be necessary in order to validate its actual effectiveness, by comparing the outcomes obtained with those achievable using traditional surgical techniques. Furthermore, a clinical trial involving the application of the technique in different conditions, identifying the types of bone defects in which it is more effective. Since it is a combined technique, it would be also indicated to demonstrate how the results are correlated to the biomaterial properties or to laser photobiomodulation.

## Ethics Statement

The authors have nothing to report.

## Consent

Written informed consent for publication of their clinical details and/or clinical images was obtained from the patient/parent/guardian/relative of the patient. A copy of the consent form is available for review by the Editor of this journal.

## Conflicts of Interest

The authors declare no conflicts of interest.

## Author Contributions

**Paolo Vescovi:** conceptualization and methodology. **Ilaria Giovannacci:** investigation and original draft preparation. **Gloria Bortolotti:** original draft preparation. **Roberta Iaria:** review and editing. **Jair Carneiro Leão:** review and editing. **Samir Nammour:** review and editing. **Carmen Mortellaro:** supervision and validation. **Alberta Greco Lucchina:** supervision and validation. **Marco Meleti:** supervision and validation.

## Funding

This research received no external funding.

## Data Availability

Data sharing is not applicable to this article as no datasets were generated or analyzed during the current study.
